# In Vitro Gene Expression Profiling of Quantum Molecular Resonance Effects on Human Endometrium Models: A Preliminary Study

**DOI:** 10.3390/genes16030290

**Published:** 2025-02-27

**Authors:** Angela Grassi, Maria Santa Rocca, Marco Noventa, Gianantonio Pozzato, Alessandro Pozzato, Marco Scioscia, Alessandra Andrisani, Giovanni Pontrelli, Carlo Foresta, Luca De Toni

**Affiliations:** 1 Veneto Institute of Oncology IOV-IRCCS, 35128 Padova, Italy; 2 Department of Medicine, University of Padova, 35128 Padova, Italy; mariasanta.rocca@aopd.veneto.it (M.S.R.); carlo.foresta@unipd.it (C.F.); 3 Unit of Gynecology and Obstetrics, Department of Women and Children’s Health, University of Padova, 35100 Padova, Italy; marco.noventa2@unipd.it (M.N.); alessandra.andrisani@unipd.it (A.A.); 4 Telea Electronic Engineering S.r.l., 36066 Sandrigo, Italy; gianantonio.pozzato@teleamedical.com (G.P.); alessandro.pozzato@teleamedical.com (A.P.); 5 Unit of Gynecological Surgery, Mater Dei Hospital, 70125 Bari, Italy; marcoscioscia@gmail.com; 6 Department of Obstetrics and Gynecology, Policlinico Hospital, 35031 Abano Terme, Italy; giovanni.pontrelli@sacrocuore.it

**Keywords:** endometrial receptivity, exogenous electromagnetic field, gene expression profiling

## Abstract

Objectives: The identification of methods to improve the endometrial receptivity (ER) is increasingly of interest. The effect of the electromagnetic field associated with Quantum Molecular Resonance (QMR) on ER was investigated here. Methods: Ishikawa cells were used to evaluate the effects of QMR both on the expression of a group of genes involved in ER, i.e., *HOXA10*, *HOXA11*, *LIF*, *ITGB3*, and *ITGAV*, and on cell toxicity. Endometrial samples were obtained from six patients during routine diagnostic procedures, four of which were subsequently used to assess the transcriptional response to QMR through microarray. Results: Compared to unexposed controls, a single exposure of Ishikawa cells to QMR for 20 min was associated with a significant and power-dependent up-regulation of all the selected ER-related genes up to 8 power units (PU). Repeated exposure to QMR, up to three consecutive days, showed a significant up-regulation of all the selected genes at power values of 4 PU, from day two onwards. Negligible cytotoxicity was observed. Gene set enrichment analysis, on microarray data of endometrial biopsies stimulated for three consecutive days at 4 PU, showed a significant enrichment of specific gene sets, related to the proteasome system, the cell adhesion, the glucocorticoid receptor, and cell cycle pathways. Conclusions: Our results suggest a possible favorable impact of QMR on ER.

## 1. Introduction

The use of assisted reproductive technologies (ARTs) has grown significantly over the past decades, particularly thanks to the implementation of new protocols of ovary stimulation [1]. In spite of this evidence, a parallel increase in pregnancy rates has not been reported by recent ART registries. Indeed, available data evaluating ART practice and outcomes over the last ten years worldwide demonstrated substantially unchanged live-birth rates, with a mean value of around 20% of babies born from either fresh or frozen embryo transfer [2,3,4]. Since healthy pregnancy relies on both embryo and maternal factors, the adequate receptivity of the endometrium represents a key aspect in order to obtain a successful embryo implantation upon fertilization [5]. To this regard, the most recent literature emphasizes a strict monitoring of endometrial parameters in order to synchronize the oocyte fertilization, or the embryo transfer, with the window of implantation (WOI). WOI is recognized as the lapse of time that generally spans between day 20 and 24 of a regular menstrual cycle, corresponding to the highest receptivity for blastocyst adhesion by the endometrium primed by ovarian steroids [6,7,8,9]. The histological evaluation of endometrium, based on morphological assessment of biopsy specimens, has been considered as the most valuable method for the clinical diagnosis of WOI since the mid-1970s [10]. However, its accuracy and clinical relevance as a predictor of endometrial receptivity (ER) was questioned in subsequent studies [11]. Interestingly, the identification of WOI has benefited by the recent development of new molecular diagnostic tools based on transcriptomic profiling in endometrial biopsies [12,13,14]. The value of these approaches as diagnostic tools to address the ER status for the personalized timing of embryo transfer is undoubted; however, available data on the clinical efficacy of these tools are still controversial, further emphasizing the multifactorial complexity of the implantation process [15,16,17]. Although the diagnosis of ER is currently under extensive investigation, less emphasis is given to the identification of specific procedures aimed at improving the receptivity of endometrial epithelium, whether pharmacological or non-pharmacological [18,19].

An increasing body of literature supports the role of exogenous electromagnetic fields (EMFs) in the regulation of cell and tissue physiology [20]. To this regard, the application of EMF was shown to improve wound healing, to reduce inflammation and to stimulate angiogenesis and the synthesis of extracellular matrix in several experimental models [21,22,23,24,25,26,27,28]. Accordingly, EMFs are used as a therapeutic application in the field of regenerative medicine. This is particularly the case of Quantum Molecular Resonance (QMR) technology, consisting of the administration of low-intensity/high-frequency (4 and 64 MHz) non-ionizing alternating electric fields, currently applied for regenerative purposes after total knee arthroplasty and showing a reduction in edema [29]. Importantly, the exposure to QMR in contraindicated in individuals with pacemakers, implanted medical devices, or metal implants because of the risk of electromagnetic interference [30]. The evaluation of the transcriptional profile in bone-marrow-derived mesenchymal stem cell stimulated with QMR showed an increased expression of genes involved in extracellular matrix remodeling, wound healing, angiogenesis, and embryogenesis [31]. Taken together, these findings suggest that QMR may influence endometrial function and receptivity.

The aim of this study was to evaluate the molecular basis underlying the biological effects of QMR on natural fertility, especially with regard to endometrial receptivity. To this end, cytotoxicity and the possible stimulating effect associated with QMR exposure were evaluated in vitro in Ishikawa cells, recognized as highly representative of human endometrial cells and widely used in numerous basic research areas, such as reproductive biology and molecular science [32]. Thereafter, experiments were translated in an ex vivo model of human endometrial biopsy.

## 2. Materials and Methods

### 2.1. QMR Exposure

QMR exposure was performed using a QMR generator device supplied by Telea (Telea Electronic Engineering, Sandrigo, VI, Italy). The QMR generator setup was as follows: power supply: 230 V~50/60 Hz; maximum power in input: 250 VA; power in output: 45 W/400 Ω. The fundamental wave was at 4 MHz and the subsequent ones increased in harmonic content until 64 MHz with related decreasing amplitudes. Harmonic components were attenuated by built-in hardware and software filters of the QMR generator. The harmonic components intensities are part of the company’s know-how and matter of patent filing. The exposure was delivered through the raise of effective powers in output (from 4 to 45 W), corresponding to an increased value of power units (PU) employed as QMR settings.

**Figure 1:**
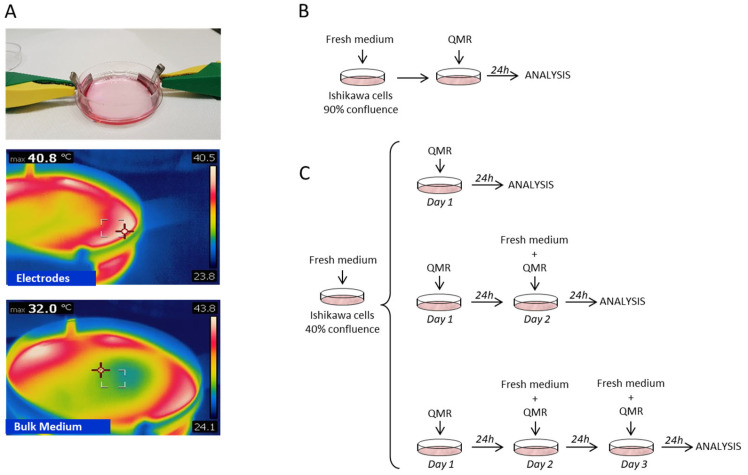
(**A**) Representative images of QMR exposure system. Cell model, cultured in 60 mm Petri dishes, received QMR exposure through two curved plate metal electrodes connected with the QMR generator by external crocodile plugs. (**B**) Experimental scheme of single day exposure to QMR and (**C**) repeated QMR exposure up to three consecutive days as detailed in the Section 2.

The QMR delivery system was composed of two curved metal electrodes of 30 mm. The electrodes were placed inside two Petri dishes, immersed in the culture media, and connected with the QMR generator by external crocodile plugs (Figure 1A).

Cells or endometrial biopsies underwent QMR exposure at different PU for daily sessions of 20 min at room temperature under the sterile environment of a biohazard hood. Such an exposure schedule was adopted according to available protocols of treatment in humans for regenerative purposes [33]. The thermal effect of QMR administration to cell cultures was evaluated by the measurement of the temperature of culture medium by a thermal camera FLIR E50 (FLIR Systems Inc. Wilsonville, OR, USA).

### 2.2. Cell Cultures and Endometrial Biopsies

Human endometrial epithelial adenocarcinoma Ishikawa cells (kind gift from Prof. Marcello Maggiolini, University of Calabria, Cosenza, Italy) were cultured in minimum essential medium with Earle’s salts and without phenol red (Invitrogen, Karlsruhe, Germany), supplemented with 2 mM L-glutamine, 1% non-essential amino acids, and 10% FBS (all from Invitrogen) at 37 °C under 5% CO_2_.

In single QMR exposure experiments (Figure 1B), cells were grown in 6 cm diameter Petri dishes up to 90% confluence, and the medium was refreshed immediately before stimulation. After QMR exposure, the conditioned medium was withdrawn, centrifuged, and stored at −80 °C to assess acute release of lactate dehydrogenase (LDH, see below). The fresh medium was then added, and cells were maintained at 37 °C in 5% CO_2_ for further 24 h of recovery. The conditioned medium was then withdrawn, centrifuged, and stored at −80 °C to assess late release of LDH, and the cells were then harvested to evaluate gene expression.

In consecutive QMR exposure experiments, the cells were grown up to 40% confluence in order to avoid excessive cell proliferation during prolonged culture conditions. The medium was refreshed immediately before QMR exposure and maintained for further 24 h of recovery. This procedure was applied to cells up to three consecutive days. After the recovery period, the conditioned medium was withdrawn, centrifuged, and stored at −80 °C to assess late release of LDH, and the cells were then harvested to assess gene expression.

The cytotoxic effect of QMR on Ishikawa cells was evaluated by the colorimetric determination of lactate dehydrogenase in conditioned medium from cells cultures with LDH cytotoxicity assay kit, according to manufacturer’s instructions (Pierce–Thermo Fisher–Life Technologies, Monza, Italy).

Endometrial samples were obtained from six patients attending the Department of Obstetrics and Gynecology, Policlinico Hospital, Abano Terme, Padova, Italy. Informed consent for the diagnostic procedure was obtained with a clearance for the anonymous use of their clinical data for scientific purposes according to the European privacy law. The endometrial biopsies were obtained during a routine hysteroscopy performed for other gynecological indications (metrorrhagia, polyps, myomas, endometriosis). The following inclusion criteria were applied: (i) ovulatory premenopausal women with regular menstrual cycle (27–33 days), (ii) no hormonal medication and intrauterine contraception use within the last 6 months before enrollment to the study. Diagnostic hysteroscopy was performed using a continuous-flow hysteroscope (Karl Storz—Karl Storz GmbH & Co., Tuttlingen, Germany) with 30° angle view optics (2.9 mm diameter) and saline solution (0.9% sodium chloride, pH 5.5) as a distension medium. Endometrial biopsies were taken with a Novak endometrial curette. Endometrial samples were portioned into two aliquots. One aliquot was fixed in 4% buffered formaldehyde for 24 h, dehydrated, embedded in paraffin, and cut into 5 mm thick sections for histological purposes (as routinely performed). The second portion not used for diagnostic purposes was washed with sterile PBS, minced into small fragments, and then divided into further two aliquots: the first underwent to QMR exposure, while the second served as unexposed control. Samples were then subjected to RNA extraction for microarray analysis as detailed below. Clinical and demographic characteristics of patients are reported in Table 1. Since the clinical history showed that patients 4 and 5 were receiving estrogen–progestin treatment, their respective biopsy samples were subsequently excluded from the analysis.

### 2.3. Real Time PCR

Total RNA from Ishikawa cells or endometrial biopsies was extracted using RNeasy^®^ Mini kit (QIAGEN, Hilden, Germany) according to the manufacturer’s instructions. Total RNA was subsequently quantified by NanoDrop UV-VIS Spectrophotometer (Thermo Fisher Scientific, Carlsbad, CA, USA). cDNA was obtained from 500 ng of total RNA by the use the SuperScript III Reverse Transcriptase. Short oligo-deoxyribonucleotide primers with random base sequences (random hexamer) were used as described by the manufacturer. cDNA was then amplified by StepOnePlus™ Real-Time PCR System (Applied Biosystem, Thermo Fisher Scientific, Monza, Italy). The total reaction volume was 10 µL and contained 2 µL of cDNA (diluited 1:5), 5 µL 2× Power SYBR Green PCR Master mix (Thermo Fisher Scientific), 1 µL of forward primer, and 1 µL of reverse primer. Primers were designed by Primer3 and are reported in Supplemental Table S1. Each reaction was performed in triplicate.

Relative quantification (RQ) was performed via the Delta Delta Ct (ΔΔCt) method [34], using GAPDH as a housekeeping gene. Differential nRQ was calculated by subtracting the expression in the corresponding control condition.

### 2.4. Microarray Analysis

To investigate the effects of QMR stimulation on human endometrium, microarray gene expression profiling was performed on four endometrial biopsies.

Total RNA was extracted from endometrial biopsies by RNeasy Mini Kit (QIAGEN, Valencia, CA, USA). DNAse treatment was performed using Ambion^®^ TURBO DNA-free™ Kit (Thermo Fisher Scientific, Carlsbad, CA, USA) according to the manufacturer’s instruction. RNA purity and concentration were assessed using NanoDrop ND-1000 (Thermo Fisher Scientific, Carlsbad, CA, USA), and RNA integrity (RIN) was determined using 4200 Agilent TapeStation System using RNA ScreenTape (Agilent, Santa Clara, CA, USA).

Synthesis and labeling of single strand (ss) cDNA from 100 ng of total RNA were performed using a WT Amplification Kit (Thermo Fisher Scientific, Carlsbad, CA, USA) according to the manufacturer’s protocol.

Successively, fragmented and labeled ss-cDNA was hybridized against the Affymetrix Human Clariom S Array (Thermo Fisher Scientific, Carlsbad, CA, USA) for 17 h at 45 °C and 60 rpm. Arrays were subsequently washed and stained with streptavidin–phycoerythrin (Gene Chip Wash and Stain Kit, Thermo Fisher Scientific, Carlsbad, CA, USA) and scanned on an Affymetrix GeneChip Scanner 3000 7G scanner.

### 2.5. Statistical Analysis

Statistical analysis was performed with SPSS 21.0 for Windows (SPSS, Chicago, IL, USA). A two-tailed Student’s t test was adopted for the comparison of continuous parameters between two groups, after assessment of normal distribution. A univariate analysis with Bonferroni–Holmes correction was adopted to compare the interaction between the power of QMR exposure and the days of consecutive exposure. Error bars in bar plots denote mean ± standard error (SE). *p*-values < 0.05 were considered statistically significant.

Bioinformatic analyses of microarray data were performed in R version 3.5.1, using the packages available in Bioconductor release 3.8. Raw data were pre-processed using the RMA algorithm as implemented in the oligo package and filtered to retain only transcript cluster ids corresponding to well-annotated genes. A differential expression analysis was carried out using the limma package, and a Benjamini–Hochberg’s method was applied to correct for multiple testing. To evaluate the significance of curated BioCarta and KEGG canonical pathways present in the Molecular Signatures Database v6.2, a Gene Set Enrichment Analysis (GSEA) was executed, ranking genes by moderated t-statistics and running the GSEA pre-ranked procedure with default parameters. Pathways with a false discovery rate (FDR) *q*-value < 0.05 were considered significant.

## 3. Results

### 3.1. Effects of the Single Exposure of Ishikawa Cells to QMR

**Figure 2:**
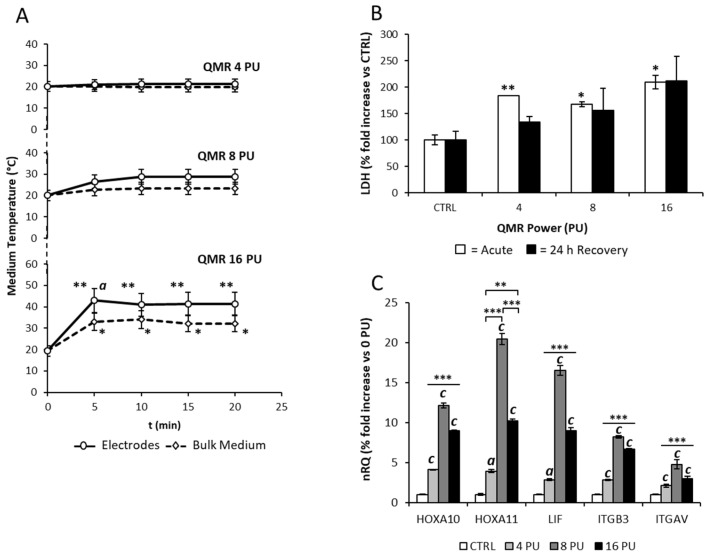
(**A**) Time/temperature profile of the cell culture system that underwent QMR exposure for 20 min at power values of 4, 8, and 16 power units (PUs). The medium temperature was monitored at both bulk medium (dashed line) and electrodes (continuous line) sites. Significance: * *p* < 0.05 or ** *p* < 0.01 vs. corresponding basal condition (time = 0 min); a *p* < 0.05 between electrodes and bulk medium sites at the indicated time point. (**B**) Lactate dehydrogenase (LDH) release in the conditioned medium from Ishikawa cells exposed to QMR for 20 min at 4, 8, or 16 PUs. The conditioned medium was sampled immediately after exposure (Acute) and after 24 h of recovery from exposure (24 h recovery). Data are reported as percentage-fold increase, with respect to the corresponding unexposed condition (CTRL). Significance: * *p* < 0.05 and ** *p* < 0.01 vs. CTRL, respectively. (**C**) Gene expression assessment of Ishikawa cells exposed to QMR for 20 min at 0, 4, 8, or 16 PU. Data are expressed as relative quantification (nRQ) of *HOXA10*, *HOXA11*, *LIF*, *ITGB3*, and *ITGAV* genes normalized on CTRL. Significance: a *p* < 0.05, b *p* < 0.01 and c *p* < 0.001 vs. CTRL, respectively; * *p* < 0.05, ** *p* < 0.01 and *** *p* < 0.001 among indicated conditions.

The possible thermal effect associated with QMR was assessed by measuring the temperature increase during the exposure to QMR. Accordingly, a 6 cm diameter Petri dish filled with the sole culture medium was exposed to QMR, at power values ranging from 4 to 16 power units (PU), up to 20 min in the sterile flow of a biohazard cap. The medium temperature was measured both in the close proximity of electrodes and within the bulk medium by the thermal camera (Figure 2A). The exposure to QMR, at either 4 or 8 PU, was not associated with a significant increase in the temperature at either site. However, exposure to the highest power of 16 PU was associated with a significant increase in the temperature, compared to the basal values. In particular, the medium temperature raised of 14 °C at the bulk medium and nearly 24 °C at the electrodes even after 5 min from the application of QMR (respectively: bulk, all *p*-values < 0.05 vs. t0; electrodes, all *p*-values < 0.01 vs. t0).

In order to assess whether the observed thermal effect associated with QMR exposure could influence cell viability, cultured Ishikawa cells underwent to the same range of QMR exposure, and the extent of cell damage was evaluated by determining the LDH released in the culture medium. To differentiate the possible acute from the late cell-damaging events associated with QMR exposure, the conditioned medium was sampled immediately after QMR exposure (acute), fresh medium was then added to cell cultures and sampled again after a 24 h recovery (Figure 2B). In the acute condition, the QMR exposure was associated with a significant increase in LDH levels in the medium, regardless of the power value of QMR. However, acute release of LDH values never exceeded the two-fold increase compared to untreated control condition.

On the other hand, LDH levels in the conditioned medium after 24 h of recovery from QMR exposure showed wide variability, and, despite a visible trend towards a direct dependence with the applied QMR power values, no significant variation was observed, compared to the unexposed control.

The expression of major genes involved in endometrial receptivity, such as HOXA10, *HOXA11*, *LIF*, *ITGB3*, and *ITGAV*, after 24 h from QMR exposure was evaluated (Figure 2C). Compared to unexposed control (CTRL), exposure to QMR was associated with a significant up-regulation of all investigated genes. In particular, gene expression increased in a power-dependent manner up to the value of 8 PU. However, at the exposure power of 16 PU, the expression of all genes showed a significant decline compared to the exposure at 8 PU. Based on this evidence, subsequent experiments were performed at power values ≤ 8 PU.

### 3.2. Effects of Repeated Exposure of Ishikawa Cells to QMR

**Figure 3:**
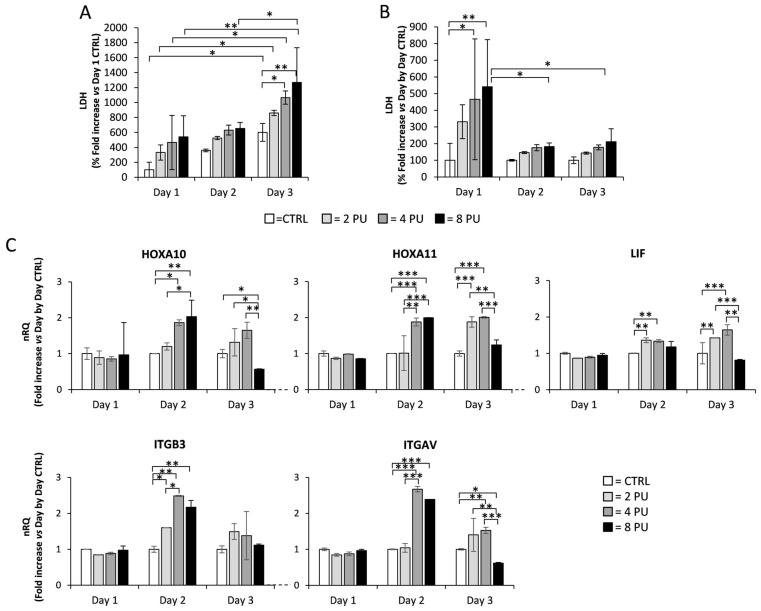
Effects of repeated exposure to Quantum Molecular Resonance (QMR) on Ishikawa cell line: (**A**) Lactate dehydrogenase (LDH) release in conditioned medium from Ishikawa cells exposed to QMR. Data are reported as percentage-fold increase with respect to unexposed control conditions (CTRL) at day one and as (**B**) percentage-fold increase with respect to the corresponding day-by-day CTRL. Significance: * *p* < 0.05 and ** *p* < 0.01 between indicated conditions. (**C**) Gene expression analysis in Ishikawa cells upon repeated exposure to QMR. Data are reported as relative quantification (nRQ) of *HOXA10*, *HOXA11*, *LIF*, *ITGB3,* and *ITGAV* genes normalized on the day-by-day CTRL. Significance: * *p* < 0.05, ** *p* < 0.01, and *** *p* < 0.001 vs. CTRL.

The approved regimen of QMR administration for regenerative purposes involves daily application for at least three successive days per week [28]. On this basis, the effect of multiple exposure to QMR was evaluated by exposing Ishikawa cells for 20 min per day to QMR, at a power value ranging from 2 to 8 PU, up to 3 consecutive days, with a recovery period of 24 h between subsequent exposures. It should be reiterated that the cells exposed to QMR had a basal confluence of 40% in order to avoid excessive cell proliferation during prolonged culture conditions. Assessment of LDH release in the conditioned medium and gene expression profiling were then performed (Figure 3).

The possible cytotoxicity deriving from multiple QMR exposure was assessed by evaluation of LDH released in the medium (Figure 3A). Despite a general trend towards an increase in LDH levels in the conditioned medium along with the increase in culture days, a significant increase in LDH levels was observed only between the corresponding exposure powers at day one and day three. Furthermore, at day three, a significant increase in LDH levels compared to the unexposed control, was observed at 4 and 8 PU, respectively. In order to address the possible role of prolonged culture conditions on LDH release, a day-by-day normalization on each control sample was performed (Figure 3B). By this approach, significant effect of QMR exposure on spontaneous cell death was found only at day one at power values ≥ 4 PU. Differently, no significant effect on cell death associated with consecutive exposure was found at day two and three.

The effect of consecutive QMR exposure on the expression of *HOXA10*, *HOXA11*, *LIF*, *ITGB3*, and *ITGAV* genes was then evaluated using a day-by-day normalization approach on control samples in order to address the possible specific effect of QMR (Figure 3C). At day one, the exposure to QMR, at any power value, was not associated with any significant variation in the expression of any assessed gene, compared to unexposed control condition. Differently, at day two, a peculiar power-dependent response pattern was found for each of the genes assessed. In particular, *HOXA10*, *HOXA11*, and *ITGAV* showed a significant upregulation upon exposure to QMR at a power value ≥ 4 PU compared to unexposed control. For these genes, exposure to QMR at 8 PU was associated with no further increase in gene expression compared with 4 PU. On the other hand, *LIF* and *ITGB3* genes showed a significant increase in expression already at 2 PU. However, whilst LIF expression was not further increased at 4 and 8 PU, the expression of *ITGB3* gene was progressively and significantly increased at 2 and 4 PU, with no significant variation at 8 PU. At day three, a significant up-regulation of *HOXA11* and *LIF* genes, compared to the control, was observed at QMR power values of 2 and 4 PU. *ITGAV* showed up-regulation only at a QMR power value of 4 PU. However, at a QMR power value of 8 PU, all the aforementioned genes showed a significant blunt in their expression. On the other hand, *ITGB3* showed no significant variation at any of the power values assessed.

Taken together, these data suggest that repeated exposure to QMR has negligible effects on cell viability and is associated with increased expression of genes involved in endometrial receptivity up to a QMR power value of 4 PU. For this reason, subsequent experiments of QMR exposure were performed at the power value of 4 PU.

### 3.3. Effects of Multiple Daily Exposure to QMR on Gene Expression in Endometrial Biopsies

**Figure 4:**
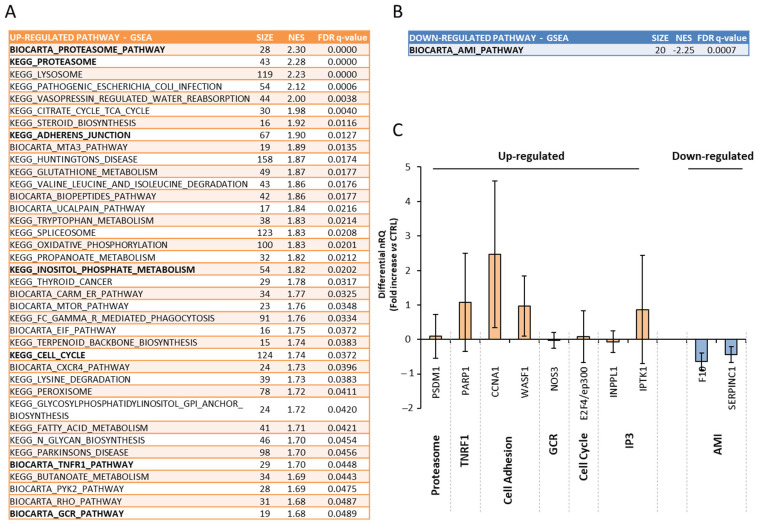
Effects of multiple daily exposure to Quantum Molecular Resonance (QMR) on gene expression in endometrial biopsies. Results of gene set enrichment analysis, performed on microarray data related to four endometrial biopsies that underwent daily exposure to QMR, at 4 power units for 20 min, for three consequent days, and four matched specimens from biopsies not exposed to QMR (controls). (**A**) reports a list of canonical pathways showing significant up-regulation after QMR exposure, while (**B**) reports those showing a significant down-regulation. For each pathway, the number of genes (size), the normalized enrichment score (NES), and false discovery rate (FDR) *q*-value are shown. (**C**) reports validation by qRT-PCR of selected genes from both up-regulated and down-regulated pathways (in bold in (**A**,**B**)). Deregulation of genes in samples exposed to QMR with respect to controls is shown in terms of differential nRQ.

In order to characterize the gene expression pattern in human endometrium in response to QMR, a microarray analysis was performed on endometrial biopsies. Samples were stimulated for three consecutive days at a power value of 4 PU for 20 min. Results were compared to four unstimulated controls (Figure 4).

Due to the heterogeneity of the analyzed samples, the differential expression analysis showed that only one gene, namely *TPD52 like 1*, was significantly up-regulated in QMR-stimulated samples with respect to controls, showing a 3.5-fold increase and a Benjamini–Hochberg adjusted *p*-value = 0.023.

A gene set enrichment analysis was thus performed to evaluate whether the QMR stimulation caused a coordinated deregulation of canonical pathways (Figure 4). Interestingly, among the significantly up-regulated pathways, we found some related to proteasome, tumor necrosis factor-receptor 1 (TNFR1), cell adhesion, glucocorticoid receptor (GCR), cell cycle, and inositol trisphosphate (IP3). In addition, only acute myocardial infarction pathway (AMI) showed to be significantly downregulated after QMR exposure. In order to validate these results, the differential expression of selected genes from the aforementioned pathways was assessed with a real-time PCR in stimulated samples compared to unstimulated controls (Figure 4C). A significant up-regulation was confirmed, respectively, in *PARP1* gene from the TNFR1 pathway, *CCNA1* and *WASF1* genes from cell adhesion pathways, and *IPTK1* gene from the IP3 pathway (respectively, *PARP1 p* = 0.048, *CCNA1 p* = 0.023, *WASF1 p* = 0.038, *IPTK1 p* = 0.049 vs. controls). On the other hand, *F10* and *SERPINC1* genes from the AMI pathway were equally and significantly downregulated in stimulated samples compared to unstimulated controls (respectively, *F10 p* = 0.013, *SERPINC1 p* = 0.018 vs. controls).

## 4. Discussion

This is the first study that provides evidence about the application and possible biological effects of QMR on the human endometrium. Specifically, we investigated the effects of QMR exposure on cell toxicity and gene expression on two in vitro models of human endometrial epithelium. Data on the Ishikawa cell line, an acknowledged model of hormone-responsive human endometrial epithelia, showed that QMR exposure up-regulates the expression of genes involved in endometrial receptivity in a power-dependent manner. Importantly, QMR showed negligible effects on cell viability. Finally, the application of gene expression profiling to endometrial biopsies exposed to QMR showed a significant up-regulation of pathways involved in key cell functions, such as cytosol remodeling, hormone receptivity, and cell adhesion. Taken together, these data suggest a positive effect of QMR as treatment for improving endometrial receptivity.

Despite the increase in couple infertility over the last decades and the increasing use of ART procedures to overcome this problem, the general effectiveness of ART is actually little encouraging, accounting for a live birth rate of approximately 20% [35]. This evidence is generally ascribable to poor oocyte and embryo quality, especially in women of late reproductive age, and to defects in endometrial receptivity. Endometrial receptivity is currently considered a major independent factor regulating all processes from embryo adhesion onwards to the onset of pregnancy [36]. Accordingly, the stimulation of endometrial receptivity is considered an effective approach to improve the overall pregnancy rate. From a pharmacological point of view, there is no current evidence supporting the use of a specific protocol of hormonal treatment associated with improved efficiency in stimulating endometrial receptivity [5,37]. On the other hand, from a diagnostic point of view, great emphasis was given to the development of microarray-based assays for the evaluation of the endometrial receptivity status [38,39] On this basis, gene expression analysis represents an appropriate tool for the evaluation of the efficacy of novel methods to improve the endometrial receptivity.

QMR is a novel technology gaining interest for its use in regenerative medicine [31,40]; however, no data are available on its possible effect on endometrial epithelia. Therefore, we first evaluated any possible toxicity effect on Ishikawa cell line cultures, showing that neither acute nor chronic QMR exposure were associated with markers of cell death. On the other hand, QMR showed a positive effect on the expression of genes related to embryo adhesion and implantation. *HOXA10*, *HOXA11*, *ITGAV*, *ITGB3*, and *LIF* genes were all shown to play a relevant role in embryo adhesion and implantation, undergoing massive up-regulation during the window of implantation and therefore considered as markers of endometrial receptivity [41,42,43,44,45,46,47,48]. Our data showed that acute stimulation with QMR was associated with a significant up-regulation of all the aforementioned genes, with a clear dependency on the power of the applied electrical field. To evaluate the possible physiological relevance of these preliminary results, an ex vivo approach was adopted by exposing endometrial biopsies to QMR. Notably, bioptical endometrial specimens are currently considered the “gold standard” to represent endometrial function [49]. Furthermore, since current molecular evaluation of endometrial receptivity for clinical aims relies on microarray-based assays, we decided for a similar approach in this study. A microarray gene expression profiling was performed to evaluate the differential activation of signaling pathways in QMR-exposed endometrial biopsies, compared to unstimulated controls. Importantly, although differential expression analysis was not able to identify significant changes in single genes in response to QMR exposure, the gene set enrichment analysis revealed a significant deregulation of genes belonging to several pathways. In particular, canonical pathways involved in the proteasome system, glucocorticoid signaling, and cell adhesion were significantly activated upon QMR exposure. To this regard, Manohar et al., using a 2-D gel electrophoresis–proteomic approach, showed that proteasome subunit α type-5, tubulin-polymerization-promoting protein family member 3, superoxide dismutase [Cu-Zn], and sorcin were significantly decreased in the mid-secretory phase in endometrium of infertile women, highlighting their physiological relevance in endometrial differentiation/maturation [50]. In addition, the observed up-regulation of pathways involved in glucocorticoids signaling agrees with the known role of these hormones on endometrial function [51]. Furthermore, deregulation of genes involved in cell adhesion pathway was associated with angiogenesis and tissue remodeling in maternal-to-zygotic transition during embryo development [52,53,54,55].

We acknowledge the preliminary nature of our findings. At the time of execution of the experiments, the study management allowed for the recruitment of a small sample size in order to address the possible effect of QMR exposure on a valuable model of human endometrium. Further studies are required to confirm these data. In addition, a limited culture timeframe is an intrinsic issue of cell models. Prolonged culture of adherent cell lines resulted in increased cell death due to catabolites accumulation [56]. On the other hand, endometrial biopsies showed an even more reduced culture life span because of tissue decontextualization. Indeed, the most reliable model to test the effect of QMR exposure would be in vivo animal models, which are beyond the scope of this study. However, taken together, our data support the safety profile of controlled QMR exposure and its favorable effect on endometrial receptivity.

## 5. Conclusions

In conclusion, here, we provide evidence that QMR exposure has a stimulating effect on endometrial function by activating key pathways involved in endometrial receptivity. Further studies are required to confirm these preliminary findings and to determine whether the application of QMR might be indicated for the clinical management of female infertility associated with endometrial factors.

## Figures and Tables

**Table 1:** Clinical and demographic characteristics of women donors of endometrium biopsies used for microarray analysis.

Donor ID	Age (Years)	Clinical Diagnosis	Pharmacological Treatment
1	42	suspected endometriosis	none
2	37	previous endometriosis	none
3	48	myomas	none
4	34	menometrorrahgia	progesterone
5	48	menometrorrahgia	estrogen–progesterone
6	42	endometriosis	none

## Data Availability

Microarray data have been deposited in the ArrayExpress database (https://www.ebi.ac.uk/biostudies/arrayexpress, accessed on 30 January 2025) under accession number E-MTAB-14820.

## Supplementary Materials

The following supporting information can be downloaded at: https://www.mdpi.com/article/10.3390/genes16030290/s1, Supplemental Table S1. Primers. Ref. [57] is cited in Supplementary Materials.
