# Supplementary material for: In Vitro Gene Expression Profiling of Quantum Molecular Resonance Effects on Human Endometrium Models: A Preliminary Study
Source: Genes (Basel). 2025 Feb 27;16(3):290. doi: 10.3390/genes16030290 (PMC11942151; doi:10.3390/genes16030290)
Supplement: Supplementary file 1 [file genes-16-00290-s001.zip › genes-3479825-supplementary.pdf]

Supplemental Table S1. Primers.

Gene	Forward	Reverse	Ref
HOXA10	5'-GGTTTGTTCTGACTTTTTGTTTCT-3'	5'-TGACACTTAGGACAATATCTATCTCTA-3'	[1]
HOXA11	5'-AGTTCTTTCTTCAGCGTCTACATT-3'	5'-TTTTTCCTTCATTCTCCTGTTCTG-3'	[1]
LIF	5'-GGAGGTCACTTGGCATTCAG-3'	5'-GGAAGAGAACGAAGAACCTACC-3'	[1]
ITGB3	5'-ACCATCTCTTTACCTCCTAATTCC-3'	5'-CTGGCTCTACAATAGCACTCTC-3'	
ITGAV	5'-AAACAGAATTTGTAAGTTGGCAGAT-3'	5'-GGTGACATTGAGATGGGTAGTG-3'	
ACTB	5'-GCAAGCAGGAGTATGACGAGT-3'	5'-CAAGAAA GGGTGTAACGCAACTAA-3'	[1]
UBA1	5'-GCGTGTCCATGCTCTATTCC-3'	5'-TCTCTGTCATCGGCTGATCC-3'	
PSMD11	5'-CCATCGTGAAGCGTGACATT-3'	5'-TCCAGTCTTTGCCAGGAGAG-3'	
SERPINC1	5'-GGCCATCAACAAATGGGTGT-3'	5'-TCCACAGGCCCTTGAAGTAA -3'	
F10	5'-CATATGATGCAGCCGACCTG-3'	5'-GCTCAGAATGGTTCCACCAC-3'	
GRIP1	5'-GCCATGACGAGATCATCAGC-3'	5'-TGACCTCCACTGTTCGGAAA-3'	
ERCC3	5'-CCGTTGCCATTAGCACCTAC-3'	5'-TCGGAACATCTTGGCTGGTA-3'	
NOS3	5'-GAGTATGACGTGGTGTCCCT-3'	5'-AGCTGCAAAGCTCTCTCCAT-3'	
PARP1	5'-AGAGTGCCAACTACTGCCAT -3'	5'-TGCCCAAACCTTTGACACTG -3'	
LMNA	5'-TAGGTGAGGCCAAGAAGCAA -3'	5'-CGCAGCTCCTCACTGTAGAT-3'	
WASF1	5'-CATGGAGCAGGAGATGCAAA-3'	5'-ACACAGGTGTTCTGCCTGTA-3'	
EP300	5'-TGCAAACAATCGAGCGGAAT-3'	5'-AGGCCCTGGATTCATGGAAA-3'	
CCNA1	5'-TGCACTTCCTGCTGGATTTC-3'	5'-GTCTGGCTGCTTCTTCATGT-3'	
IPTK1	5'-AGAGGCCCTCACTCAAGAAC-3'	5'-TTTGACACGTTGTGGCTGTT-3'	
INPPL1	5'-CCCACGCTCAAACCAATTCT-3'	5'-GTGCTGCCGATCATGGATTT-3'	
PSMD1	5'-GAGGAGGAACAAGAGCCAGA-3'	5'-GCAGGTCTCGGCATGAATTT-3'	
E2F4	5'-GGACCCAACCCTTCTACCTC-3'	5'-GGGCAAACACTTCTGAGGAC-3'	
GNAS	5'-ACCAAGTTCCAGGTGGACAA-3'	5'-GCAGTCACATCGTTGAAGCA-3'	
GAPDH	5’-AAGGTGAAGGTCGGAGTCAA-3’	5’-AATGAAGGGGTCATTGATGG-3’	

Reference

1. Celik, O.; Unlu, C.; Otlu, B.; Celik, N.; Caliskan, E. Laparoscopic Endometrioma Resection Increases Peri-Implantation Endometrial HOXA-10 and HOXA-11 MRNA Expression. *Fertil Steril* 2015, *104*, 356–365, doi:10.1016/j.fertnstert.2015.04.041.
